# Immune history confers antibody- and T cell-dependent cross-protection against highly pathogenic avian influenza H5N1 viruses

**DOI:** 10.1128/jvi.02088-25

**Published:** 2026-01-22

**Authors:** Pamela H. Brigleb, Bridgett Sharp, Lauren Lazure, Brandi Livingston, Shelby Patrick, Victoria Meliopoulos, Ericka Kirkpatrick Roubidoux, Lee-Ann Van de Velde, Shaoyuan Tan, Dorothea R. Morris, Tyler Ripperger, Lauren Rowland, Alexis C. Thompson, Katie Kleinhenz, Velmurugan Balaraman, Kiril Dimitrov, Paul G. Thomas, Stacey Schultz-Cherry

**Affiliations:** 1 Department of Host-Microbe Interactions, St Jude Children's Research Hospital 5417, Memphis, Tennessee, USA; 2 Texas A&M Veterinary Medical Diagnostic Laboratory 14741, Canyon, Texas, USA; 3 Texas A&M Veterinary Medical Diagnostic Laboratory 117328 , College Station, Texas, USA; Dartmouth College Geisel School of Medicine, Hanover, New Hampshire, USA

**Keywords:** transmission, CD4 T cells, immune-mediated protection, antibodies, bovine outbreak, highly pathogenic avian influenza viruses

## Abstract

The outbreak of highly pathogenic avian H5 influenza (HPAI) clade 2.3.4.4b in cattle has spread across the United States. Mice with pre-existing immunity to H1N1 virus or with a live-attenuated influenza vaccine showed protection against a lethal bovine-derived HPAI H5N1 viral challenge. Notably, ferrets with mixed immunity also demonstrated protection against a feline-derived H5N1 virus, independent of cross-reactive neutralization titers, but antibodies to whole virus were observed. To investigate protective factors, we conducted T cell epitope mapping using published H1N1 viral sequences and found high conservation of key T cell epitopes in the bovine HPAI H5N1 strain. Depletion of T cells in mice prior to and during primary H1N1 infection impacted cross-protective antibodies to H5N1 virus, with CD4 depletion increasing mortality and CD8 depletion mildly impacting morbidity upon H5N1 viral challenge. This underscores the need to investigate memory T cell responses alongside antibodies in assessing preexisting cross-protection to HPAI H5N1 viruses.

**IMPORTANCE:**

The rapid spread of highly pathogenic avian H5 influenza (HPAI) clade 2.3.4.4b in U.S. cattle represents an urgent and evolving public health threat. Our findings reveal that pre-existing immunity, whether from seasonal H1N1 infection or live-attenuated vaccination, can confer substantial protection against lethal bovine- and feline-derived HPAI H5N1 viruses, even in the absence of strong cross-neutralizing antibody titers. By integrating T cell epitope mapping with mechanistic depletion studies, we demonstrate that conserved CD4 and CD8 T cell epitopes across H1N1 and H5N1 strains underpin this cross-protection. Critically, loss of CD4 T cell help during primary H1N1 infection disrupts the development of cross-reactive antibody responses and markedly worsens outcomes after H5N1 challenge. These results identify memory T cell responses as important determinants of heterosubtypic immunity and highlight the need to incorporate T cell-focused metrics into risk assessment, vaccine evaluation, and preparedness strategies for emerging HPAI H5N1 viruses.

## INTRODUCTION

In March 2024, highly pathogenic avian influenza (HPAI) H5N1 virus clade 2.3.4.4b was discovered in dairy cows in the United States, with spillover events to mammals including cats and humans (1–3). By November 2025, this strain had been identified in 18 states, as reported by the United States Department of Agriculture (4). Since the outbreak started, there have been 70 confirmed human cases primarily in farm workers, with people presenting with generally mild-to-moderate clinical symptoms (5–7). Previous spillover events with HPAI H5Nx viruses in humans have an average fatality rate of around 50% (8), and with the increasing number of case reports in humans and multiple avenues of crossover events to other mammals (2), it is important to understand mechanisms of protection against HPAI H5N1 clade 2.3.4.4b viruses.

A recent study found that persons born after the 1980s have little-to-no neutralizing antibody responses to bovine HPAI H5N1 virus (9, 10). This corroborates previous findings that the elderly may have higher number of antibodies that are cross-reactive to H5N1 viruses (11). In ferrets, prior H1N1 virus infection immunity with a 2009 pandemic strain provided protection against lethal challenge of bovine HPAI H5N1 clade 2.3.4.4b virus independent of H5N1-specific neutralizing antibodies (9, 10, 12). This is corroborated by our previous findings in mice that prior infection with the H1N1 A/Puerto Rico/08/34 (PR8) virus was sufficient for protection, and cross-reactive antibodies to H5N1 whole virus were found (13). These results indicate there are factors that contribute to protection against HPAI H5N1 viral infection independent of neutralizing antibodies, such as non-neutralizing antibodies and memory T cell responses, at least with older H1N1 influenza strains.

In this study, we tested whether prior influenza exposure through the 2009 H1N1 virus or vaccinated with the 2023–2024 FluMist live-attenuated vaccine (LAIV) had protective immunity against HPAI H5N1 clade 2.3.4.4b viral infection in mice. We further tested whether ferrets with mixed pre-existing immunity, vaccination with an LAIV, and/or H1N1 infection were protected against lethal challenge of an HPAI H5N1 clade 2.3.4.4b virus isolated from a cat. Pre-existing immunity in both mice and ferrets provided some level of protection against lethal HPAI H5N1 challenge. Protection occurred despite the absence of cross-protective hemagglutination inhibition (HAI) or microneutralization antibodies and was instead associated with cross-reactive antibodies to whole virus.

To explore the potential contribution of T cells to protection, we conducted *in silico* analyses of previously defined influenza T cell epitopes. We found several conserved regions between H1N1 and the H5N1 viral challenge strains, suggesting that memory T cells elicited by prior infection or vaccination may cross-recognize H5N1 antigens, a possibility that warrants further investigation. *In vivo* depletion studies showed that loss of CD4 or CD8 T cells during primary H1N1 infection impaired cross-protective antibody responses and exacerbated disease to H5N1 challenge. Together, these findings suggest that cross-reactive non-neutralizing antibodies and T cells contribute to cross-protective immunity to H5N1 viruses. Collectively, our study highlights the importance of immune history in shaping responses to emerging HPAI H5N1 viruses and highlights the need to consider immune mechanisms beyond HAI responses in evaluating population risk.

## RESULTS

### Prior H1N1 virus infection history or vaccination with LAIV provides mild to high protection against lethal bovine HPAI H5N1

**Fig 1:**
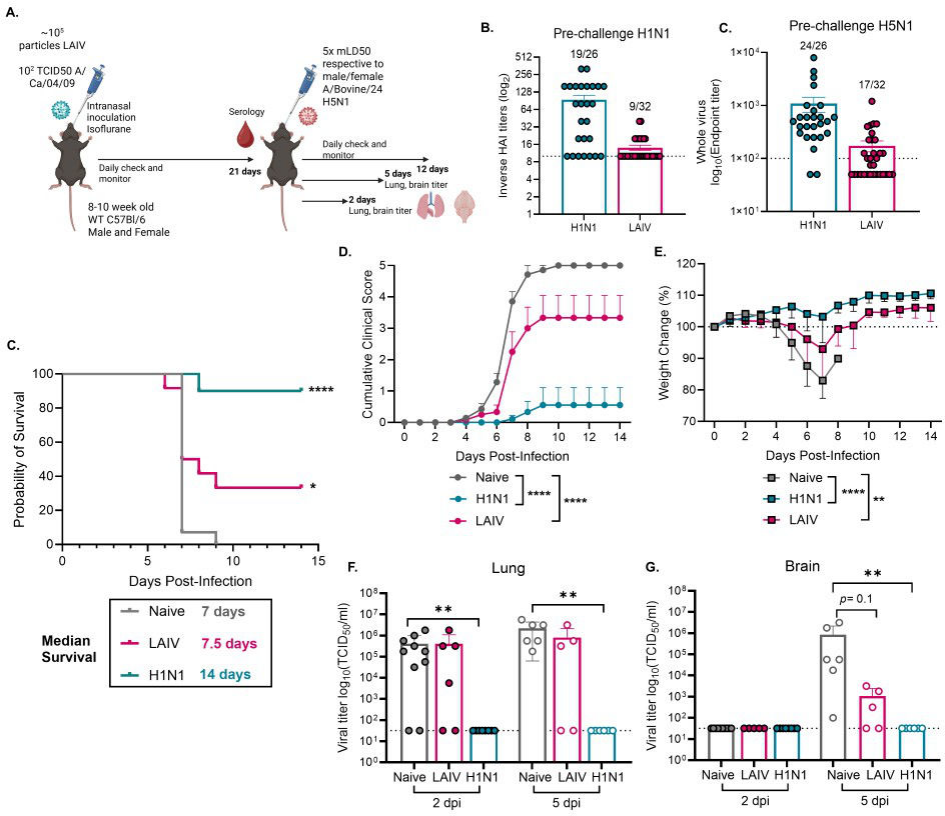
Prior H1N1 infection or LAIV vaccination confers protection against lethal bovine-derived H5N1 viral challenge. (**A**) Graphical summary of experimental design made in *Biorender*. (**B**) Pre-challenge serology against H1N1 (HAI titer) or H5N1 (whole-virus ELISA titer) viruses in mice with immune history. The numbers represent how many out of the total mice were positive for each assay, and only mice that were positive for one or both assays were included in the following experiments. (**C–E**) (*n* = 9–10) Mice were infected with 5× respective mLD_50_’s (male and female) of A/bovine/Ohio/24 by IN inoculation and were monitored for survival (**C**), clinical scores (**D**), and weight loss (**E**) or (**F, G**) mice were sacrificed at 2 or 5 dpi, and lung (**F**) and brain (**G**) tissues were assessed for viral titers by TCID_50_ (*n* = 5–10). Statistical analyses include log-rank Mantel Cox test (**C**), two-way analysis of variance (ANOVA) with multiple comparisons (**D, E**), and Kruskal-Wallis test with Dunn’s multiple comparisons (**F, G**) *P < 0.05; **P < 0.01; ****P < 0.0001. Data are shown as mean with standard error of mean.

We have previously demonstrated that prior infection with the H1N1 A/Puerto Rico/08/34 (PR8) virus protected mice from lethal HPAI clade 2.3.4.4b bovine H5N1 infection (13). We were curious whether a more contemporary H1N1 strain, A/California/04/2009 (CA/09) virus, or an LAIV from the previous influenza season (FluMist, 2023-24) would also provide protection against lethal bovine HPAI H5N1 viral challenge. To test, a combination of adult male and female wild-type (WT) C57BL/6J mice were intranasally (IN) inoculated with a mild infection of 100 50% tissue culture infectious dose (TCID_50_) of CA/09 or ~10^5^ particles of LAIV under light anesthesia. At 3 weeks post-infection, pre-challenge sera was collected, and mice were challenged with 5× their respective mean lethal-dose 50 (mLD_50_) (13) of the bovine HPAI H5N1 virus to A/bovine/Ohio/B24OSU-439/2024 (A/bovine/Ohio/24) virus. The mice were split into three groups: two for tissue collection for viral titers on days 2 and 5 post-infection and one for weight loss, clinical scores, and survival (Fig. 1A). Pre-challenge sera were assessed for both HAI titers to H1N1 viruses (CA/09 and A/Victoria/2022 for LAIV) and for H5N1 antibodies by whole virus enzyme-linked immunosorbent assay (ELISA). This was selected since we and others have previously reported poor HAI detection and responses against bovine H5N1 infection (13). Most mice had either a positive H1N1 pre-challenge HAI titer (HAI titer ≥ 1:20) or a positive antibody response to H5N1 whole virus (≥ 1:100), and those that did not were excluded from the study as a lack of response likely reflected ineffective infection or vaccination (Fig. 1B).

We found that prior infection with the H1N1 CA/09 virus or vaccination with LAIV provided significantly high to moderate protection against mortality from lethal bovine HPAI H5N1 virus, with 90% protection with H1N1 virus immune history and 40% protection with LAIV, compared to 0% survival in naïve mice (Fig. 1C). We also observed protection against morbidity, with significantly decreased cumulative clinical scores (Fig. 1D) and decreased weight loss (Fig. 1E) compared to the naïve group. Strikingly, there were no detectable viral titers in the lung and brain at days 2 and 5 post-infection in the H1N1 virus immune history group (Fig. 1F and G). While there were still detectable viral titers in the LAIV immune history group (Fig. 1F and G), we detected a reduction in brain viral titer at 5 days post-infection (dpi) compared to the naïve group (Fig. 1G). Overall, immune history from an H1N1 virus infection or LAIV vaccination provided some level of protection against mortality and morbidity from lethal bovine H5N1 viral challenge.

### Mixed pre-existing immunity provides protection against direct lethal challenge of a feline-derived HPAI H5N1 clade 2.3.4.4b virus

**Fig 2:**
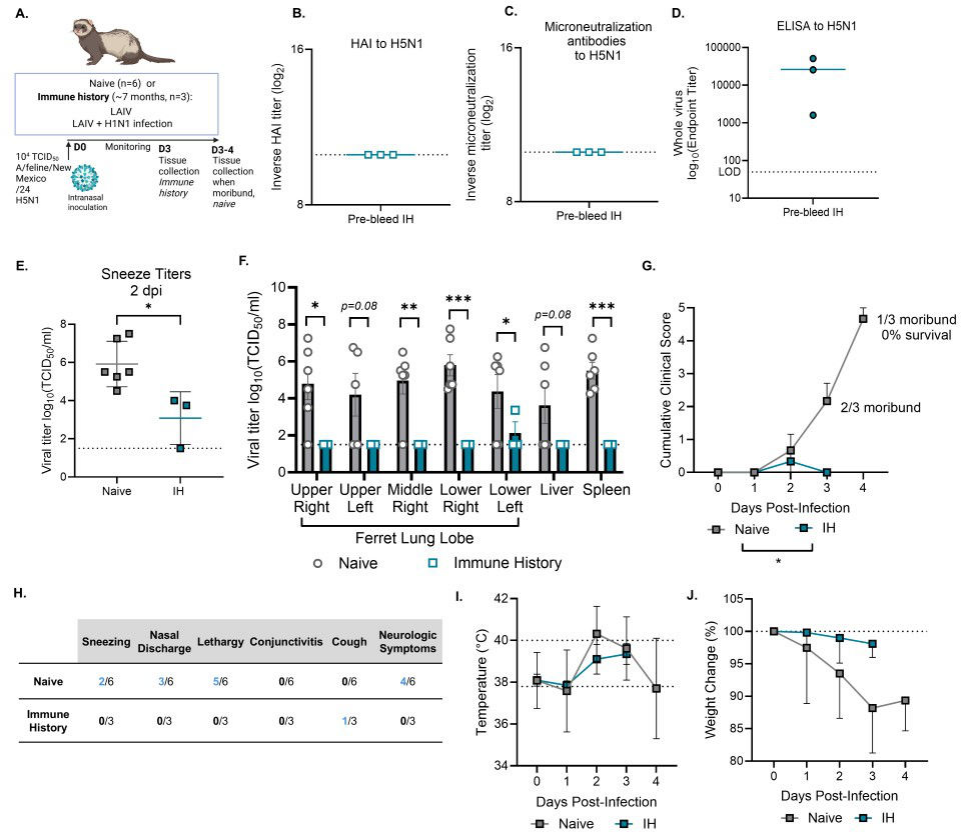
Mixed pre-existing immunity protects ferrets from lethal challenge with feline-derived HPAI H5N1 virus. (**A**) Graphical summary of experimental design made in *Biorender*. Ferrets were IN inoculated with 10^4^ TCID_50_ of A/Feline/NewMexico/24 and monitored daily (*n* = 3–6 ferrets/group). Naïve ferrets were monitored daily until moribund when tissues were collected for titers, and immune history ferrets were sacrificed at 3 dpi to compare disease severity and viral loads. (**B**) Pre-bleed HAI titers to H5N1; (**C**) microneutralization titers to H5N1; or (**D**) whole-virus ELISA titers to H5N1; (**E**) titers determined by TCID_50_ in sneezes at 2 dpi. (**F**) Viral loads as determined by TCID50 in multiple lung lubes and systemic tissues at time of sacrifice. (**G**) Weight change (%) over time. (**H**) Body temperature. (**I**) Description of clinical scores in ferrets, with the shown number of ferrets with that clinical score throughout the study out of the total number of ferrets in that group. (**J**) Summation of the total clinical score daily. Statistical analyses done with Student’s t test with Welch’s correction (**E and F**) and a mixed-effects model (**G, H, J**). *P < 0.05; ** P < 0.01; ***P < 0.001. Data are represented as mean with standard deviation.

We isolated a clade 2.3.4.4b HPAI H5N1 from a cat found deceased near a dairy farm in New Mexico, A/feline/NewMexico/F001/2024 (A/feline/NewMexico/24). This strain is similar to but not identical to the A/bovine/Ohio/24 virus used in the murine immune history studies (Table 1) and had overall higher replication in primary ferret nasal epithelial cells compared to the bovine-derived H5N1 strain (14). Since no studies to date have investigated whether immune history in ferrets also provides protection against feline-derived HPAI H5N1 viruses, we IN inoculated naïve (*n* = 6) or immune history ferrets (*n* = 3) with 10^4^ TCID_50_ of A/feline/NewMexico/24 H5N1 and monitored them daily (Fig. 2A). Immune history ferrets (ferret ID numbers 4476, 4498, and 5001, Methods) were previously vaccinated with an LAIV, the FluMist 2009–2010 season, or an H1N1/H3N2 LAIV with a PR8 backbone currently being developed at St. Jude ~6 months previously followed by CA/09 H1N1 viral challenge.

**TABLE 1:** Amino acid sequence similarity among influenza A strains compared to A/bovine/Ohio/B24OSU-439/2024 including A/feline/NewMexico/F001/2024, A/Puerto Rico/8/1934, A/California/04/2009, A/Victoria/4897/2022, A/Wisconsin/67/2022, A/Ann Arbor/6/60, and A/Norway/31694/2022

	Bovine vs feline H5N1	Bovine vs PR8	Bovine vs CA/09	Bovine vs Victoria	Bovine vs Wisconsin	Bovine vs Ann Arbor	Bovine vs Norway
PB2	99.87%	95.52%	96.57%	95.65%	95.65%	94.99%	95.52%
PB1	99.87%	96.57%	96.96%	96.70%	96.70%	98.68%	96.70%
PA	99.72%	95.81%	96.37%	95.53%	95.67%	94.97%	95.67%
NP	100%	93.98%	93.98%	93.78%	93.98%	93.37%	93.98%
HA	100%	64.22%	62.61%	61.78%	62.13%	71.55%	61.95%
NA	100%	83.58%	89.77%	86.35%	86.14%	45.40%	86.14%
M1	100%	92.46%	92.86%	91.27%	91.27%	93.25%	91.67%
M2	100%	83.51%	88.66%	86.60%	86.60%	81.44%	86.60%
NS1	99.13%	91.30%	79.13%	76.09%	76.52%	85.65%	76.52%
Avg % similarity (including HA)	99.82%	88.55%	88.55%	87.08%	87.18%	84.37%	87.19%
Avg % similarity (no HA)	99.82%	91.59%	91.79%	90.25%	90.32%	85.97%	90.35%

We found that immune history ferrets had no cross-reactive HAI or microneutralization responses to H5N1 pre-challenge but did have cross-reactive antibodies to whole H5N1 virus measured by ELISA (Fig. 2B through D). In naïve ferrets, direct infection with A/feline/NewMexico/24 H5N1 virus caused 100% mortality by day 4 post-infection. This was associated with high sneeze titers at 2 dpi (Fig. 2E) and viral loads in the lungs with systemic spread (Fig. 2F), weight loss, elevated body temperature, and clinical scores including neurological symptoms prior to humane euthanasia (Fig. 2G through J). In contrast, immune history ferrets were protected from HPAI A/feline/NewMexico/24 H5N1 viral challenge up until the scheduled sacrifice at 3 dpi. Since the majority of naïve ferrets had succumbed to infection by this time, immune history ferrets were euthanized at 3 dpi to enable direct comparison of viral replication at a matched disease stage. Leading up to that point, immune history ferrets had significantly lower sneeze titers at 2 dpi (Fig. 2E), reduced viral loads in the lungs and in systemic tissues (Fig. 2F), only mild clinical signs with only one of three having a cough and none developing neurological disease (Fig. 2G and H)**,** and minimal weight loss and change in body temperature (Fig. 2I and J). Together, these findings demonstrate that mixed immune history protects ferrets from lethal H5N1 challenge by limiting viral replication and disease severity.

### Direct-contact (DC) transmission of feline-derived HPAI H5N1 is lethal in naïve ferrets but limited by immune history

**Fig 3:**
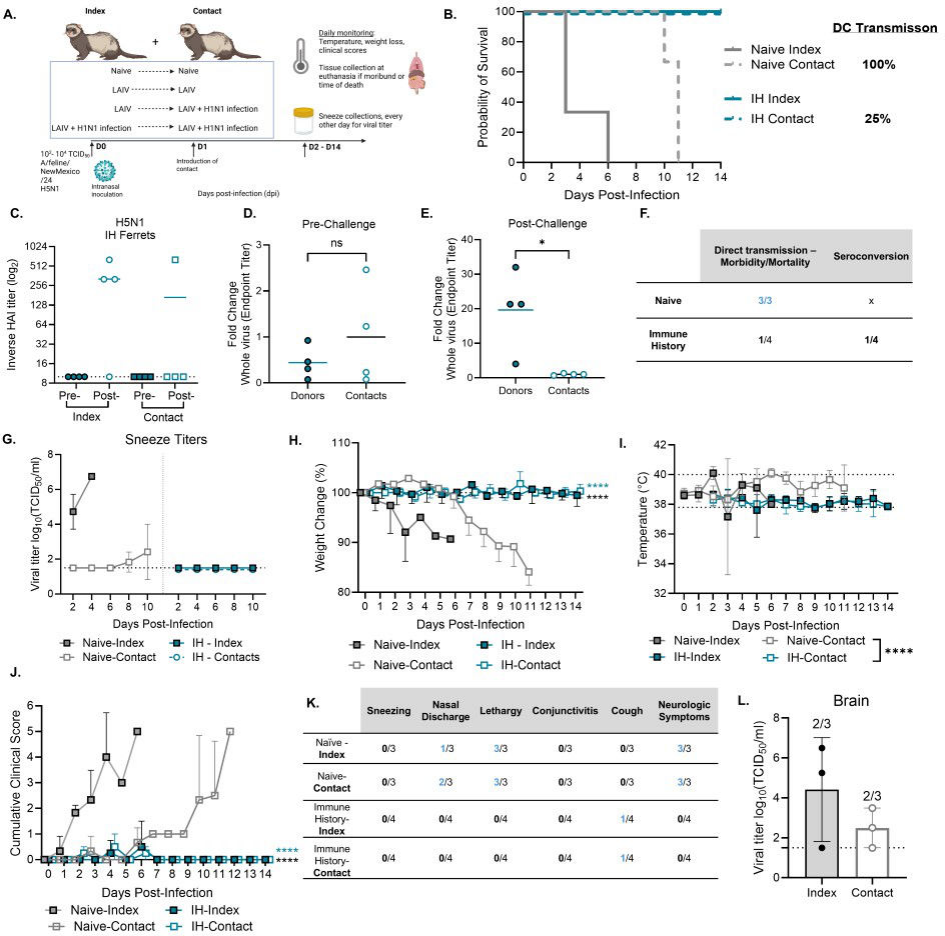
DC transmission of feline-derived HPAI H5N1 virus is lethal in naïve ferrets but limited by pre-existing immunity. (**A**) Graphical summary of the experimental design made in *Biorender*. Naïve (*n* = 3) or mixed immune history (*n* = 4) index ferrets were IN inoculated with 10^2^–10^4^ TCID_50_ of A/feline/NewMexico/24 and monitored daily. After 24 h, the naïve contact (*n* = 3) or mixed immune history (*n* = 4) contacts were introduced for DC throughout the completion of the study. (**B**) Survival and summary of DC transmission in naïve or immune history pairs. Pre-and post-challenge bleeds were assessed for H5N1 HAI titers (**C**) or (**D**) pre- or (**E**) post-challenge H5N1 whole-virus ELISA antibody titers. (**F**) Summary of mortality in the direct transmission model and seroconversion, which was assessed by HAI responses (**G**) Sneeze titers of index and contacts over time every other day; (**H**) weight change (%); (**I**) body temperature; (**J**) cumulative clinical scores; (**K**) description of clinical scores in ferrets, with the shown number of ferrets with that clinical score throughout the study out of the total number of ferrets in that group. (**L**) Brain titers from naïve index or contact ferrets at the time of sacrifice, with the numbers representing how many out of the total ferrets had detectable virus in the brain. Statistical analyses include log-rank Mantel Cox test (**B**), an unpaired t test with Welch’s correction (**D, E**), and a two-way ANOVA with multiple comparisons (**H–J**). ns = not significant; *P < 0.05; **** *P* < 0.0001. Data are represented as mean with standard deviation.

To test whether our feline-derived HPAI H5N1 virus transmits by DC and whether immune history modifies transmission and disease outcomes, we cohoused naïve (*n* = 6) or immune history contact ferrets (*n* = 8) with their corresponding index ferrets at 1 dpi with A/feline/New Mexico/24 virus until the conclusion of the study at 14 dpi (Fig. 3A). Immune history ferrets had been vaccinated with either the 2010–2011 season FluMist or an H1N1/H3N2 LAIV with a PR8 backbone currently in development at St. Jude, followed by challenge with phosphate-buffered saline (PBS) or CA /09 H1N1 virus ~6 months prior to the start of these studies (see Methods for detailed information; index ferret IDs are 4477, 4497, 4499, and 5002=2; contact ferret IDs are 4476, 4498, 4500, and 5001). Contact immune history ferrets that did not undergo seroconversion to H5N1 by HAI or an increase in whole-virus ELISA titers were repurposed for Fig. 2.

All naïve index-contact pairs succumbed to infection, indicative of 100% DC transmission and lethality (Fig. 3B). In contrast, immune history index-contact pairs survived, indicating 100% protection from mortality in both directly infected and contact ferrets (Fig. 3B). To assess cross-protective serological responses, we measured H5N1 HAIs and whole-virus ELISAs pre- and post-challenge or exposure. As expected, we did not observe cross-reactive HAI responses pre-challenge; only post-H5N1 exposure was observed (Fig. 3B). As pre-bleeds were collected ~6 months prior to H5N1 challenge, direct comparisons with post-challenge titers were not directly informative. Therefore, ELISA responses were normalized to those of the contact ferrets to assess relative fold changes. Using this approach, we observed a clear increase in the fold change of cross-reactive antibody responses in index immune-history ferrets post-challenge compared to the contact ferrets (Fig. 3C). These results revealed that only one out of four contact immune history ferrets underwent seroconversion to H5N1, indicative of transmission, compared to 100% transmission and lethality observed in the naïve controls.

Furthermore, in the naïve index ferrets, we observed high sneeze titers at 2 and 4 dpi, with sneeze titers present in the naïve contacts at 8 and 10 dpi. We also observed substantial weight loss, changes in body temperature, and high cumulative clinical scores including nasal discharge, lethargy, and neurological symptoms in both naïve index and contacts. Viral titers were also detected in the brain in two out of three naïve index and contacts at the time of sacrifice. In contrast, immune history ferrets did not have detectable virus in the sneezes over time, minimal weight and body temperature changes, and only mild clinical scores with one of four having a cough post-ferret sneezing. Collectively, these results indicate that immune history provides a level of protection against transmission and corresponding mortality, in contrast to the naive controls.

### Identifying factors that may contribute to protection against HPAI H5N1 viruses

We have shown that prior infection with H1N1 and LAIV vaccination offers protection against lethal HPAI H5N1 clade 2.3.4.4b challenges. However, HAI antibodies do not appear to correlate with this protection. Here, we demonstrate that immune-history mice exhibit cross-reactive antibodies to HPAI H5N1 prior to challenge, as measured by whole-virus ELISA (Fig. 1 to 3).

We compared the amino acid sequences of viral proteins from the feline H5N1 virus and several influenza A viruses represented in our immune-history studies—CA/09, PR8, and A/Ann Arbor/6/60 and A/Norway/31694/2022 (FluMist)—or viruses included in the seasonal influenza vaccine: A/Victoria/4897/2022 and A/Wisconsin/67/2022 (Table 1). Since the feline H5N1 isolate was highly similar to our bovine H5N1 isolate, exhibiting 99.82% sequence homology, we conducted the remainder of our studies with the bovine H5N1 virus strain.

Unsurprisingly, the most variable viral protein compared to the bovine H5N1 virus was HA, with the closest similarity being 71.55% to the H2N2 A/Ann Arbor/6/60 virus (Table 1). However, there is moderate conservation of the NA protein across HXN1 strains, with the highest similarity of 89.77% being to the CA/09 H1N1 virus (Table 1). Upon conducting amino acid sequence alignments with other viral proteins, we noted a high conservation of internal proteins, including nucleoprotein (NP) and matrix protein 1 (M1), as well as RNA polymerase proteins such as PB1 and PB2 (Table 1). Several key T cell epitopes critical for protective responses against H1N1 viruses are known to reside within NP, PB1, and M1 (15–18). T cell memory responses to influenza are crucial for protection, alongside antibody responses. Given the high conservation of viral proteins containing well-characterized T cell epitopes, we next explored the potential for eliciting protective T cell immunity in our immune-history groups against HPAI H5N1 viral challenge.

### High conversation of previously mapped T cell epitopes of influenza A viruses

**Fig 4:**
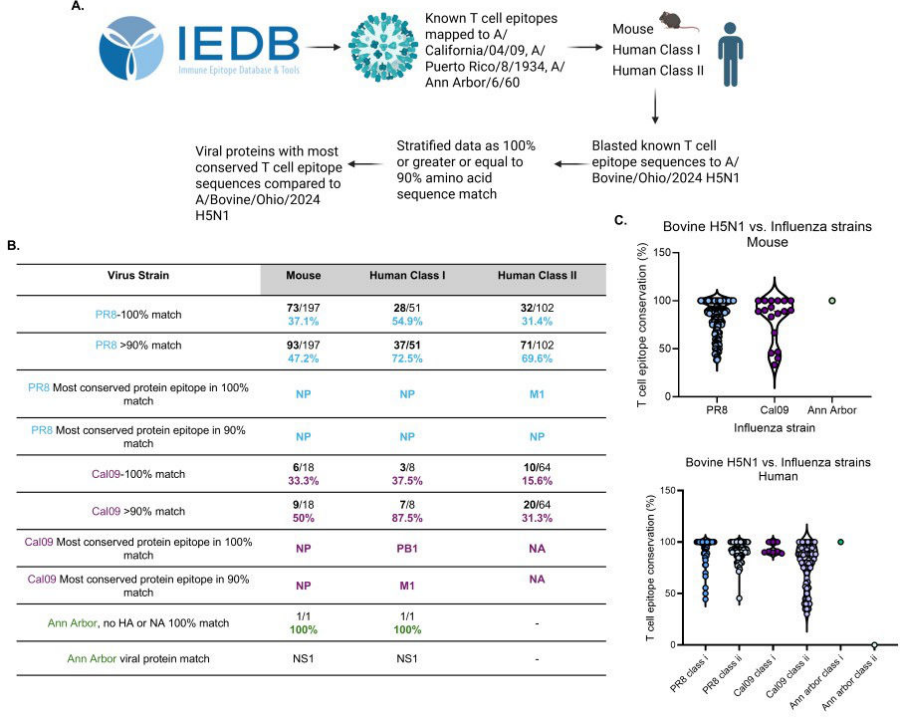
Conservation of previously mapped T cell epitopes between bovine-derived H5N1, H1N1, and H2N2 strains. (**A**) Graphical summary of workflow made in *Biorender* using Immune Epitope Database and Tools (**B**) Summary table of percent of conserved T cell epitopes from PR8, CA/09, or Ann Arbor/60 in the A/bovine/Ohio/2024 HPAI H5N1 virus and (**C**) percent conserved for each known mapped T cell epitope for mouse and human, including both class I and class II restricted T cell epitopes.

To assess whether previously mapped T cell epitopes from other influenza A viruses have the potential to provide cross-protection against HPAI H5N1 virus, we utilized the Immune Epitope Database & Tools interface (Fig. 4A). We identified a total of 350 T cell epitopes for PR8 virus (Table S2, S5 and S6), 90 epitopes for CA/09 virus (Tables S3, S7, and S8), and one for Ann Arbor/60 virus (Table S4), which we then separated based on mouse, human class I, or human class II restricted known epitopes. We then employed the Epitope Conservancy Analysis from the Immune Epitope Database to determine whether each known T cell epitope amino acid sequence was conserved in the bovine HPAI H5N1 virus. We categorized our results based on the level of amino acid conservation: 100% or ≥90% (averaging one amino acid dissimilarity). Notably, the NS1 protein human and mouse T cell epitope mapped for Ann Arbor/60, the backbone of FluMist LAIV, was conserved across the bovine H5N1 strains (Fig. 4B).

For epitopes with ≥ 90% conservation, we observed a T cell epitope conservancy of 43.2% between PR8 and bovine H5N1 in mice and 72.5% and 69.6% conservancy in human class I and class II, respectively (Fig. 4B and C). The most conserved T cell epitopes for the PR8 virus were associated with the NP viral protein (Fig. 4B, Table S2). While fewer T cell epitopes were mapped for the CA/09 virus, we noted similar trends to those of the PR8 virus, albeit with decreased overlap in human class II epitopes (Tables S6 and S8). In contrast to PR8 epitopes conserved in bovine H5N1 viruses, the most conserved epitopes across the range of viral proteins included NP, PB1, M1, and NA (Fig. 4B).

Several T cell epitopes, including NP366 and PB1703 (17), are recognized as dominant correlates of protection following PR8 influenza infection in mice (16, 19). Furthermore, there are several immunodominant and validated human class I T cell epitopes that are important in protection against influenza challenge (16). Our analysis confirmed that the amino acid sequences of these immune-dominant T cell epitopes are highly conserved in the bovine HPAI H5N1 strain from both mice and human CD8 T cell epitopes (Table 2). Additionally, we compared the amino acid sequences of the conserved T cell epitopes (≥90% similarity) in bovine HPAI H5N1 with modern circulating H1N1 strains and those included in seasonal or 6+2 FluMist vaccines. We found a broad conservation of these sequences across H1N1 strains, ranging from 72.02% to 100% (Tables S1, S10, and S11).

**TABLE 2:** Conservation of influenza virus human CD8 T cell epitopes in the HPAI H5N1 clade 2.3.4.4b strains used in these studies (A/bovine/Ohio/24 and A/feline/NewMexico/24), other H5 clades, and H7N9 viruses

Epitopes	Sequence	Clade 2.3.4.4b		Clade 1	Clade 1.1	Clade 2.1.1	Clade 2.2.1	Clade 2.3.2.1a	H7N9	
		A/bovine/Ohio/24	A/feline/New Mexico/24	A/Vietnam/1203/04	A/Cambodia/R0405050/07	A/duck/Hunan/795/02	A/turkey/Turkey/1/05	A/duck/Bangladesh/17D1012/18	A/Guangdong/17SF003/2016	A/HongKong/125/17
M1_58–66_	GILGFVFTL	100%	100%	100%	100%	100%	88.9%	100%	100%	100%
NP_145–156_	DATYQRTRALVR	100%	100%	100%	100%	100%	100%	100%	100%	100%
NP_265–273_	ILRGSVAHK	100%	100%	100%	100%	100%	100%	100%	100%	100%
NP_338–346_	FEDLRVLSF	88.9%	88.9%	88.9%	88.9%	88.9%	88.9%	88.9%	88.9%	88.9%
NP_380–388_	ELRSRYWAI	100%	100%	100%	100%	100%	100%	100%	100%	100%
NP_383–391_	SRYWAIRTR	100%	100%	100%	100%	100%	100%	100%	100%	100%
PB1_413–421_	NMLSTVLGV	100%	100%	100%	100%	100%	100%	100%	100%	100%
PB1_498–505_	RYGFVANF	100%	100%	100%	100%	100%	100%	100%	100%	100%

These findings suggest that the high conservation of known T cell epitopes from both historical and currently circulating H1N1 viruses, including immunodominant epitopes, has the potential to play a role in the protection against HPAI H5N1 clade 2.3.4.4b viruses, in addition to the non-neutralizing antibodies we detected (Fig. 1). Collectively, these studies underscore the importance of exploring additional factors that may contribute to protection against HPAI H5N1, including T cells.

### T cells support cross-protective immune responses elicited by H1N1 pre-existing immunity to H5N1 infection

**Fig 5:**
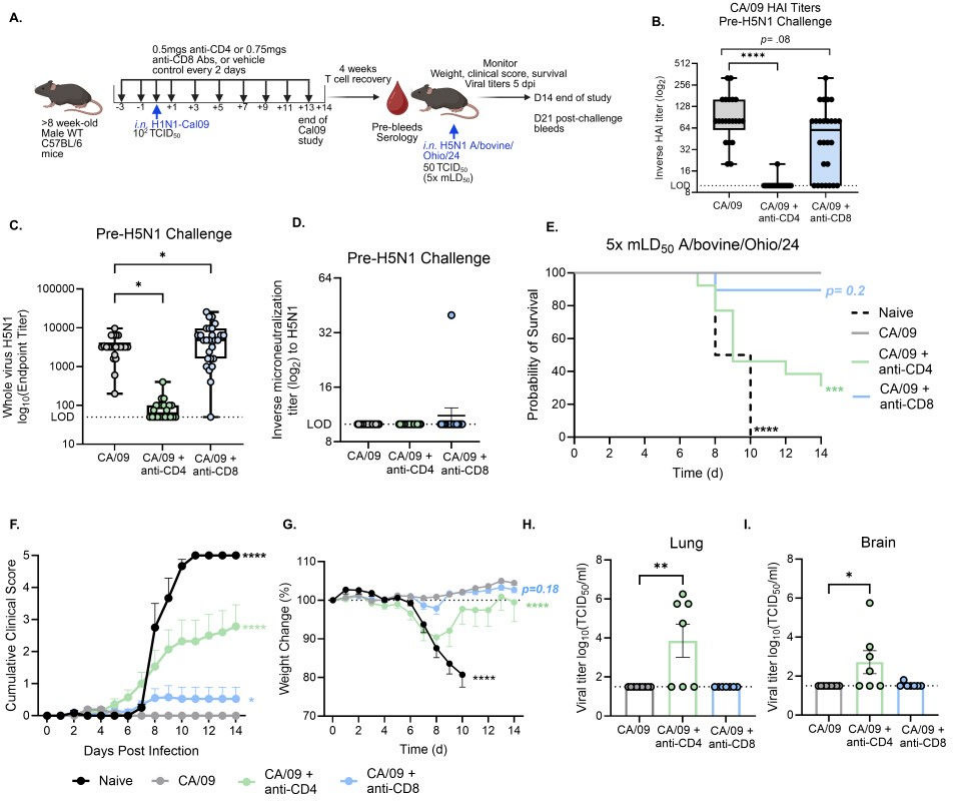
T cell-mediated contribution to immune history-mediated protection against H5N1 virus. (**A**) Graphical summary of experimental design made in *Biorender*. Mice were administered an anti-CD4- or anti-CD8-depleting antibody or a vehicle control prior to and during infection with 10^2^ TCID_50_ CA/09 (**B**) Pre-challenge sera 4 weeks post T cell depletion, and H1N1 infection was assessed for CA/09 H1N1 HAI titers, (**C**) H5N1 whole-virus ELISA antibody titers and (**D**) microneutralization titers to H5N1. (**E–I**) Mice were challenged with 5× mLD_50_ (50 TCID_50_) of A/bovine/Ohio/24 H5N1 and monitored daily for (**E**) survival and (**F**) clinical scores and (**G**) weight loss over time. At 5 dpi, lung and brain titers were assessed by TCID_50_ assay (**H and I**). Statistical analyses include one-way ANOVA with Dunnett’s multiple comparisons test (**B, C, H, I**), Log-rank Mantel-Cox test (**E**), and a two-way ANOVA with Dunnett’s multiple comparisons test (**F, G**) *P < 0.05; **P < 0.01; ***P < 0.001; ****P < 0.0001. Data are shown as mean with standard error of mean.

To determine the contribution of CD4 and CD8 T cells to H1N1-mediated protection against H5N1, we depleted these subsets *in vivo* prior to and during primary infection, and depletion was confirmed in blood and lung tissue. Mice were inoculated with a mild dose of 10^2^ TCID_50_ of CA/09 virus and were monitored through 14 dpi. We then waited 4 weeks to allow for recovery of respective T cells, at which point pre-challenge sera were collected to assess antibody responses (Fig. 5A). Vehicle-treated mice infected with CA/09 virus developed robust HAI titers to CA/09 (Fig. 5B) and cross-reactive whole-virus ELISA antibodies to H5N1 (Fig. 5C). CD8 depletion reduced CA/09 HAI titers, although not significantly, but these mice maintained or even showed increased cross-reactive whole-virus H5N1 ELISA titers (Fig. 5B and C). This suggests that CD8 T cells contribute to the development of homologous HAI responses and may also influence the type and quality of antibody responses that cross-react with H5N1 viruses (Fig. 5B and C). In contrast, CD4 depletion significantly blunted both CA/09 HAI titers and cross-reactive whole-virus H5N1 ELISA titers to H5N1, highlighting the essential role of CD4 T cells in generation of protective antibody responses (Fig. 5B and C). Notably, little-to-no cross-protective microneutralization titers to bovine-derived H5N1 virus were detected in CA/09 pre-existing immunity mice (Fig. 5D).

Four weeks post-conclusion of primary H1N1 CA/09 infection and antibody depletion (14 dpi), mice were challenged with 5× LD_50_ (50 TCID_50_) as previously determined (*13*) of A/bovine/Ohio/24 H5N1. Consistent with previous findings (Fig. 1), vehicle-treated mice infected with CA/09 immune history were protected against lethal H5N1 viral challenge (Fig. 5E)**,** with only mild clinical scores (Fig. 5F) or weight change (Fig. 5G) and no detectable viral titers in the lungs or brain at 5 dpi (Fig. 5H and I)**,** while naive controls exhibited complete mortality with severe disease including neurological symptoms and high viral loads (Fig. 5E through I).

CD8-depleted mice showed mildly reduced survival and increased clinical scores, despite having little-to-no detectable virus in tissues, suggesting a modest influence on disease outcomes, although not significant (Fig. 5E through I). In contrast, CD4-depleted mice had significantly reduced survival, worsened clinical scores, and 4/7 mice had detectable viral titers in the lungs and brain, consistent with the loss of CD4-dependent antibody responses and potential direct antiviral CD4 memory T cell functions (Fig. 5E through I). Collectively, these findings indicate that CD4 T cells, and to a lesser extent, CD8 T cells contribute to immune history-mediated protection against H5N1, with CD4 T cells critical for antibody-dependent protection and CD8 T cells contributing modestly to limiting disease severity.

## DISCUSSION

As human infections with bovine-related HPAI H5N1 viruses continue to emerge, it is crucial to identify factors that mediate protection and determine the role of pre-existing influenza immunity against these viruses. Recent studies have demonstrated that prior H1N1 immunity can confer protection against bovine-derived HPAI H5N1 virus in ferrets (9, 10, 12) and mice (13). However, our findings indicated that cross-reactive HAI and microneutralization antibodies to HPAI H5N1 virus are not associated with pre-existing protective immunity, highlighting the need for further investigation. This study aimed to explore the protective effects of the pandemic H1N1 CA/09 virus and/or LAIV against HPAI bovine and cat-derived H5N1 viruses (clade 2.3.4.4b) using both murine and ferret models, with the goal of identifying potential factors that contribute to protection against lethal viral challenges. Notably, we identified cross-reactive antibodies targeting the whole H5N1 virus. Future studies should further delineate whether these cross-reactive antibodies are targeting the HA, including HA1 or HA2, or NA.

Comparative amino acid sequence analysis revealed only ~ 64.22% and 62.21% homology between the HA of HPAI H5N1 and PR8 and CA/09 viruses, respectively, with greater conservation observed in the NA protein (83.58% similarity to PR8 and 89.77% to CA/09). This suggests that the cross-protective antibodies we identified may be directed against the NA protein, which was also observed in recent studies (12). Given the high conservation of NA among H1N1 strains, including modern circulating viruses and those in seasonal influenza vaccines, it is probable that prior infection with contemporary influenza strains provides some degree of protection against newly emerged HPAI H5N1 clade 2.3.4.4b viruses, which warrants further investigation.

The recent approval of the LAIV FluMist for home use in the United States raises questions about its effectiveness against HPAI H5N1 viruses. In our studies, we utilized three different LAIV formulations: the 2023–2024 season FluMist in mice, the 2010–2011 season FluMist, and a PR8 backbone LAIV containing seasonal H1N1 and H3N2 proteins under pre-clinical development at St. Jude. While vaccination with FluMist alone conferred mild protection against lethal HPAI H5N1 challenges in mice, the combination of LAIV with prior H1N1 infection resulted in robust protection in ferrets challenged with a feline-derived HPAI H5N1 isolate, which may possess greater crossover potential than bovine derivatives. This suggests that LAIV vaccination, especially in conjunction with pre-existing immunity, could enhance anti-H5N1 memory responses, which is a promising avenue for further investigation.

Memory T cell responses play a pivotal role in mediating protection to influenza viruses (16, 17, 19–23). T cells also play a pivotal role in the development of antibodies during vaccination or in response to infection (20–23). We analyzed known T cell epitopes mapped for H1N1 viruses and assessed their conservation in our HPAI H5N1 strain. Our analysis revealed several conserved potential T cell epitopes, particularly in internal viral genes. Vaccination with an H5N1-specific LAIV demonstrated an increase in T cell responses to internal proteins, including matrix protein (M) and NP, thereby boosting pre-existing influenza A T cell responses from prior infections and seasonal vaccines (15). This may be an effective strategy as the majority of these top conserved T cell epitopes were also found in other H5 clade viruses and H7N9 viruses (Table 2).

We further depleted CD4 or CD8 T cells during a primary H1N1 infection to investigate their contribution to cross-protective immunity against HPAI H5N1 viral challenge. CD4 depletion significantly reduced both H1N1 HAI titers and cross-reactive H5N1 whole-virus antibodies, highlighting their essential role in supporting antibody generation and cross-protection. CD8 depletion also caused a reduction in H1N1 HAI titers. Notably, some CD8-depleted mice without detectable H1N1 HAI responses still generated cross-reactive H5N1 antibodies and survived H5N1 challenge, indicating that H1N1 HAI titers are not reliable correlates of cross-protection to A/bovine/Ohio/24 H5N1 virus. While CD8 depletion did not significantly alter mortality, it was associated with mildly increased morbidity, suggesting that memory CD8 T cells may contribute to limiting disease severity rather than survival outcomes, which warrants further investigation. Taken together, these findings highlight that CD4 T cells indirectly support cross-protective antibody responses, whereas CD8 T cells may play more direct roles in disease control. However, the precise balance of direct versus indirect contributions remains unresolved. Previous research found that human pre-existing T cell responses from healthy donors can recognize HPAI H5N1 viral epitopes (24). Future studies employing epitope mapping or peptide pools from H5N1 clade 2.3.4.4b will be needed to dissect memory T cell-specific mechanisms of protection in our pre-existing immunity animal models. Collectively, our results emphasize that both non-neutralizing antibodies and T cell responses are an important component of immune history-mediated protection against HPAI H5N1.

In summary, our findings demonstrate that immune history generated by prior H1N1 infection or LAIV vaccination provides meaningful protection against emerging clade 2.3.4.4b HPAI H5N1 viruses in both mice and ferret models. This protection occurred in the absence of cross-reactive neutralizing antibody responses in mice and ferrets. Instead, this protection was associated with cross-protective non-neutralizing antibody responses mediated by CD4 T cells and suggests both a direct and indirect T cell-mediated protective immune response. These results underscore that conventional correlates of protection, such as HAI titers, may underestimate the breadth of immunity conferred by prior influenza exposure. While our study was limited to controlled infection and vaccination in animal models, the findings suggest that diverse immune pathways, including memory T cell responses, should be considered in evaluating population susceptibility to HPAI H5N1 spillover. Further dissection of these mechanisms, including mapping conserved epitopes and testing vaccine formulations that enhance both antibody and T cell immunity, will be critical to guiding pandemic preparedness strategies.

## MATERIALS AND METHODS

### Animal husbandry

Mice were kept under controlled conditions with 12-h light/dark cycles at a temperature of 68°F (20°C) and 45% humidity, with continuous access to food and water. Ferrets were housed at ambient temperature (68°F [20°C] and 45% relative humidity) with 12-h light cycles and provided species- and study-appropriate enrichment activities. Humane euthanasia was performed in accordance with American Veterinary Medical Association guidelines when necessary, either due to humane or experimental endpoints. Indicators for humane endpoints included a loss of more than 30% of body weight and/or clinical scores of 3 or higher, which typically involved neurological symptoms such as circling, tremors, and reduced mobility. Ferrets were humanely euthanized via cardiac injection of Euthasol (Patterson Veterinary Supply).

### Biosafety

Experiments involving highly pathogenic avian influenza strains were conducted in a biosafety level 3 enhanced containment laboratory. Researchers wore appropriate respiratory protection (RACAL, Health and Safety Inc.) and personal protective equipment. Mice were kept in negative pressure, HEPA-filtered isolation containers. Infections or vaccinations with the pandemic California 2009 H1N1 and FluMist were conducted at biosafety level 2 prior to transferring to biosafety level 3.

### Cells, viruses, and vaccines

Madin-Darby canine kidney (MDCK) cells (American Type Culture Collection, CCL-34) were maintained in Dulbecco’s minimum essential medium (Corning) with 2 mM GlutaMAX (Gibco) and 10% fetal bovine serum (HyClone), at 37°C with 5% CO_2_. A/bovine/Ohio/B24OSU-439/2024 and A/California/04/2009 (H1N1) viruses were provided by Robert Webster and the Webby lab at St. Jude Children’s Research Hospital. Our lab isolated H5N1 viruses from the brain and lung of an infected cat found deceased in Curry County, New Mexico, which were used in our ferret infectivity studies (A/feline/NewMexico/F001/2024). Viruses were propagated in the allantoic cavity of 10-day-old specific-pathogen-free embryonated chicken eggs at 37°C. Allantoic fluid was clarified by centrifugation and stored at −80°C. Viral titers were assessed using TCID_50_ assays. The following reagent was obtained through BEI Resources, NIAID, NIH: FluMist Influenza Vaccine Live, Intranasal Spray, 2010–2011 Formula, NR-21987. FluMist from the 2023–2024 season was obtained from the pharmacy at St. Jude. Children’s Research Hospital.

### Mouse lethal dose 50 determination

Male and female C57BL/6J mice, aged 10 to 12 weeks, were lightly anesthetized with isoflurane and IN inoculated with 10^1^, 10^2^, or 10^3^ TCID50 of A/Ohio/24 H5N1 in a 25 μL volume (2–4 mice per dose). Mice were monitored for 13 days for changes in body weight, clinical scores, and survival. Mice were humanely euthanized if they lost more than 30% of body weight and/or had clinical scores of 3 or above, including neurological symptoms such as circling, tremors, and impaired motility.

### Viral titer determination

Viral titers were determined by TCID_50_ assays. Briefly, confluent MDCK cells were infected in duplicate or triplicate with 10-fold dilutions of tissue homogenates. For mice, tissues were homogenized by bead beating in 1 mL PBS, centrifuged for 5 min at 1,500 × *g*, and supernatants were transferred to a fresh tube. For ferrets, lung tissues were finely minced with scissors before adding 1 mL of sterile PBS and bead-beaten. Samples were then centrifuged for 5 min at 1,500 × *g* prior to measuring viral titers by TCID_50_ assay. Samples were serially diluted in minimal essential medium (MEM) plus 0.75% bovine serum albumin and 1 μg/mL tosylsulfonyl phenylalanyl chloromethyl ketone-treated trypsin (H1N1 samples only). After 3 days of incubation at 37 °C and 5% CO_2_, 50 μL of the supernatant was combined and mixed with 50 μL of 0.5% packed turkey red blood cells diluted in PBS for 45 min at room temperature and scored by hemagglutination endpoint. Infectious viral titers were calculated using the Reed-Muench method (25).

### Mouse viral infectivity

Mice were IN inoculated with 10^2^ TCID50 of CA/09, ~10^5^ particles LAIV, or were naive followed by 5× mLD_50_ of A/bovine/Ohio/B24OSU-439/2024 3 weeks later. They were monitored daily for 12–14 days for changes in body weight, clinical scores, and survival. Mice showing more than 30% weight loss and/or clinical scores above 3 were humanely euthanized. Clinical signs were scored from 0 (no signs) to 5 (death). Neurological signs, including circling and tremors, were also observed and used as criteria for euthanasia. Post-euthanasia, lungs and brains were collected and stored at −80°C for future analysis.

### Ferret viral infectivity

Ferrets were lightly anesthetized using 4% inhaled isoflurane and subsequently inoculated with a virus diluted in PBS (Corning, 21-040-CV) that contained penicillin (100 U/mL) and streptomycin (100 μg/mL; Corning 30-002 CI). The viral doses administered were 10^2^ or 10^4^ TCID_50_ of A/Feline/NewMexico/F001/2024 (H5N1). Weight and body temperature were monitored daily. Clinical assessments involved a point system to evaluate the presence and severity of symptoms. Metrics included sneezing (none = 0, mild = 1, and excessive = 2), coughing (absent = 0; present = 1), nasal discharge (absent = 0; present = 1), conjunctivitis (absent = 0; present = 1), and lethargy (active and playful = 0, active when stimulated = 1, and not active when stimulated = 2). Mild sneezing was classified as one to two occurrences during the observation period, while continuous sneezing was labeled as excessive. Clinical evaluations were conducted by at least two researchers. Nasal washes were graded as follows: clear = 0, cloudy = 1, mucus present with discoloration = 2, and thickened mucus present = 3. Results are presented as the total score.

### Ferret nasal wash collection

Ferrets received intramuscular anesthesia using ketamine (30 mg/kg; Patterson Veterinary Supply), and sneezing was triggered by administering 1 ml of PBS containing penicillin (100 U/mL) and streptomycin (100 μg/mL) dropwise into their nostrils. Samples were collected in sterile specimen cups, briefly centrifuged, and stored at −80°C until analysis. Viral titers were assessed using the TCID_50_ assay as described above.

### Transmission experiments

Individually housed ferrets were lightly anesthetized with 4% inhaled isoflurane and inoculated with the specified virus diluted in 500 μL of PBS. The ferrets receiving direct inoculation are referred to as index ferrets. After a 24-h period, a contact ferret was introduced into the same cage as the index ferret, allowing for unrestricted interaction for 14 days. Weight, body temperature, and clinical symptoms were monitored as previously described. Nasal washes were collected every 48 h, and sera were collected at 21 days post-conclusion of the experiment to assess viral transmission.

### Antibody quantification

Whole virus (H5N1) ELISAs were conducted using 384-well flat-bottom MaxiSorp plates (ThermoFisher) coated with 10^6^ TCID_50_ of the virus stock of A/bovine/Ohio/B24OSU-439/2024 overnight at 4°C. Plates were washed four times with PBS containing 0.1% Tween-20 (PBS-T) using the AquaMax 4000 plate washer system for H1N1 ELISAs or handwashed for H5N1 ELISAs. Plates were blocked with PBS-T containing 0.5% Omniblok non-fat milk powder (AmericanBio) and 3% goat serum (Gibco) for 1 h at room temperature. The wash buffer was removed, and plates were tapped dry. Mouse sera were diluted 1:5 in PBS and ran in duplicate. Positive and negative mouse sera were used as controls for both sets of ELISAs. Samples were incubated at room temperature for 2 h and then washed 4 times with PBS-T. Anti-mouse peroxidase-conjugated IgG secondary antibody was diluted at 1:3,000 (Invitrogen 62-6520) in blocking buffer, and 15 μL was added per well and incubated at room temperature for 1 h. Plates were washed 4 times with PBS-T and developed using SIGMAFAST OPD (Sigma-Aldrich) for 10 min at room temperature. Plates were read at 490 nm using a BioTek Synergy2 plate reader and Gen5 (v3.09) software. For each plate, an upper 99% confidence interval of blank wells OD values was determined and used in determining the endpoint titers. Alternatively, mouse sera were treated with receptor-destroying enzyme (Seiken 370013), and HAI assays or microneutralization assays were performed as described (26, 27) using turkey blood for H1N1 HAI assays or horse blood for H5N1 HAI assays.

### Immune history

#### Mice

Adult female and male WT C57BL/6J mice were inoculated by the IN route with ~10^5^ particles of LAIV FluMist 2023–2024 in 25 µL, or 10^2^ TCID_50_ of A/California/04/09 H1N1 in 25 µL and monitored daily for 14 dpi. At 3 weeks post-infection, mice were bled via eye bleeds prior to oral gavage to determine antibody titers or prior to challenge with lethal A/bovine/Ohio/B24OSU-439/2024.

#### Ferrets

Neutered, de-scented male ferrets were obtained from Triple F Farms (Elmyra, NY) at the age of 6 weeks. Index ferret IDs are 4477, 4497, 4499, and 5002; contact ferret IDs are 4476, 4498, 4500, and 5001. Contact immune history ferrets that did not undergo seroconversion to H5N1 by HAI or have a boost in whole-virus ELISA titers were repurposed for Fig. 2 (ferret ID numbers 4476, 4498, and 5001). The ferrets' immune history and timeline are shown in Table 3, with the time shown in days.

**TABLE 3:** Ferret immune history and timeline

Ferret no.	Sex	Vaccination	Infection strain	Time post-vaccination (days)	Time post-infection (days)	Positive microneutralization titers?
4476	Male	St. Jude LAIV	A/Cal/04/09	196	167	Yes
4477	Male	St. Jude LAIV	A/Cal/04/09	196	167	Yes
4497	Male	FluMist 10-11 season	A/Cal/04/09	196	167	Yes
4498	Male	FluMist 10-11 season	A/Cal/04/09	196	167	Yes
5001	Male	FluMist 10-11 season	A/Cal/04/09	196	167	Yes
4499	Male	St. Jude LAIV	X	196	167	Yes
4500	Male	St. Jude LAIV	X	196	167	Yes
5002	Male	St. Jude LAIV	X	196	167	Yes

### Pairwise alignment, identity, and mutation detection

Pairwise amino acid sequence alignments were conducted in R with the pairwiseAlignment function from the pwalign package. Sequence identity was then determined using the pid function available at Bioconductor (https://bioconductor.org/packages/pwalign).

### T cell epitope mapping and analysis

Influenza amino acid sequences were obtained through UniProt or GenBank. Known T cell epitopes were obtained through the Immune Epitope Database and Tools (https://www.iedb.org/) (28) by searching for selected influenza A virus strains with restrictions on mouse, human class I, or human class II but no disease restriction. These epitopes, viral protein information, and amino acid sequence range were then used in creating tables using Microsoft Excel, and the sequences were converted into FASTA files. The T cell epitopes were then compared against the known amino acid sequences for A/bovine/Ohio/B24OSU-439/2024 using the Epitope Conservancy Analysis from the Immune Epitope Database, and the percent similarity for each sequence was reported.

### T cell *in vivo* depletions

For *in vivo* depletion of CD4^+^ and CD8^+^ lymphocytes, mice received intraperitoneal injections of 0.5 mg purified, lipid-absorbed CD4 ascites (clone GK1.5, Harlan Sprague-Dawley, lot #200074), or 0.75 mg CD8 ascites (clone 2.43, Harlan Sprague-Dawley, lot #70088), or vehicle control every 2 days until the study endpoint. Depletion was verified by flow cytometry using anti-CD4 (clone RM4-5) or anti-CD8 (clone 53-6.7) (Fig. S1).

### Statistical analysis

Animals were randomly selected for control and experimental groups. Some experiments had a small sample size due to animal availability and limited access to BSL3 facilities. Statistical analyses include log-rank Mantel-Cox test, two-way ANOVA with multiple comparisons, Kruskal-Wallis test with Dunn’s multiple comparisons, Student's t-test with Welch’s correction, a mixed-effects model, and a one-way ANOVA with Dunnett’s multiple comparisons. Data are reported as mean with either standard deviation or standard error of mean. Graphs and statistical analyses were performed using GraphPad Prism version 10.0. Sample sizes and statistical methods are detailed in the figure legends.

## Data Availability

Data generated in this study are provided in the main paper, figures, supplemental figures, and tables. GenBank accession numbers for the strain A/feline/New Mexico/F001/2024 are as follows: HA, PV026111.1; NA, PV026110.1; PB1, PV026114.1; PB2, PV026112.1; PA, PV026113.1; NP, PV026115.1; M1, PV050262.1; NS1, PV026109.1.

## SUPPLEMENTAL MATERIAL

Supplemental material jvi.02088-25-s0001.docx Figure S1; Tables S1 to S11.

ASM does not own the copyrights to Supplemental Material that may be linked to, or accessed through, an article. The authors have granted ASM a non-exclusive, world-wide license to publish the Supplemental Material files. Please contact the corresponding author directly for reuse.
